# Multi-source connectivity as the driver of solar wind variability in the heliosphere

**DOI:** 10.1038/s41550-024-02278-9

**Published:** 2024-05-28

**Authors:** Stephanie L. Yardley, David H. Brooks, Raffaella D’Amicis, Christopher J. Owen, David M. Long, Deb Baker, Pascal Démoulin, Mathew J. Owens, Mike Lockwood, Teodora Mihailescu, Jesse T. Coburn, Ryan M. Dewey, Daniel Müller, Gabriel H. H. Suen, Nawin Ngampoopun, Philippe Louarn, Stefano Livi, Sue Lepri, Andrzej Fludra, Margit Haberreiter, Udo Schühle

**Affiliations:** 1 Department of Mathematics, Physics and Electrical Engineering, Northumbria University, Newcastle Upon Tyne, UK; 2 Department of Meteorology, University of Reading, Reading, UK; 3 Department of Space and Climate Physics, UCL Mullard Space Science Laboratory, Holmbury St Mary, UK; 4 Donostia International Physics Center (DIPC), San Sebastián, Spain; 5 Department of Physics & Astronomy, George Mason University, Fairfax, VA USA; 6 INAF-Istituto di Astrofisica e Planetologia Spaziali, Rome, Italy; 7 School of Physical Sciences, Dublin City University, Dublin, Ireland; 8 LESIA, Observatoire de Paris, Université PSL, CNRS, Sorbonne Université, Univ. Paris Diderot, Sorbonne Paris Cité, Meudon, France; 9 Laboratoire Cogitamus, Paris, France; 10 Climate and Space Sciences and Engineering, University of Michigan, Ann Arbor, MI USA; 11 European Space Agency, ESTEC, Noordwijk, The Netherlands; 12 Institut de Recherche en Astrophysique et Planétologie, Toulouse, France; 13 RAL Space, UKRI STFC Rutherford Appleton Laboratory, Chilton, UK; 14 Physikalisch-Meteorologisches Observatorium Davos, World Radiation Center, Davos Dorf, Switzerland; 15 Max-Planck-Institut für Sonnensystemforschung, Göttingen, Germany; 16 Present Address: Southwest Research Institute, San Antonio, TX USA

**Keywords:** Solar physics, Space physics

## Abstract

The ambient solar wind that fills the heliosphere originates from multiple sources in the solar corona and is highly structured. It is often described as high-speed, relatively homogeneous, plasma streams from coronal holes and slow-speed, highly variable, streams whose source regions are under debate. A key goal of ESA/NASA’s Solar Orbiter mission is to identify solar wind sources and understand what drives the complexity seen in the heliosphere. By combining magnetic field modelling and spectroscopic techniques with high-resolution observations and measurements, we show that the solar wind variability detected in situ by Solar Orbiter in March 2022 is driven by spatio-temporal changes in the magnetic connectivity to multiple sources in the solar atmosphere. The magnetic field footpoints connected to the spacecraft moved from the boundaries of a coronal hole to one active region (12961) and then across to another region (12957). This is reflected in the in situ measurements, which show the transition from fast to highly Alfvénic then to slow solar wind that is disrupted by the arrival of a coronal mass ejection. Our results describe solar wind variability at 0.5 au but are applicable to near-Earth observatories.

## Abstract (web summary)

Solar wind is highly structured yet variable. Close-up observations of the solar atmosphere reveal that the changing connectivity of multiple sources in the solar corona drives the observed complexity and variability in the inner heliosphere.

## Main

The European Space Agency (ESA)/NASA Solar Orbiter (SO)^1^ and NASA’s Parker Solar Probe^2^ missions are designed to determine the sources and drivers of the solar wind, a main goal of heliospheric physics. These recent missions provide inner heliospheric solar wind measurements together with unprecedented close-up, never-seen-before views of the solar atmosphere, which have revealed new phenomena, such as magnetic field reversals known as ‘switchbacks’, which could be related to solar wind origin and release processes^3–7^.

Fast solar wind (>500 km s^−1^) originates and escapes along open magnetic fields rooted in coronal holes (CHs), but slow solar wind sources (≲500 km s^−1^) and the mechanisms that release, accelerate and transport plasma into the heliosphere are poorly understood^8–10^. Fast wind is characterized by high bulk speeds, high proton temperatures, low densities, low ion charge state ratios and low electron temperatures^11,12^, consistent with CH sources. Apart from Alfvénic fluctuations^13^, the fast wind plasma parameters are smooth and continuous. Conversely, the slow wind is highly variable, with lower proton temperatures, increased proton densities, high ion charge state ratios and high electron temperatures, suggesting a hotter coronal origin. The picture is complicated, however, as slow wind can exhibit Alfvénic fluctuations^14–16^, and these can dominate particular solar cycle phases^17–19^.

Open-closed magnetic field boundaries (such as active region (AR) edges^20–22^), CH boundaries^23,24^, small low-latitude CHs^25^ and helmet streamers^26–28^ are promising slow wind source candidates. Plasma may be released into the heliosphere by interchange reconnection at open-closed field boundaries^29–31^ or across regions of rapidly changing magnetic field connectivity: separatrices or quasi-separatrix layers^32^ as in the S-web model^33^. Other proposed non-reconnection release mechanisms are based on plasma escaping through the expansion of coronal loops^34,35^.

Past studies used plasma composition diagnostics to trace solar wind from 1 au back to its source, by linking in situ measurements from the Advanced Composition Explorer to outflows at AR boundaries observed by Hinode^36,37^. However, there are no studies on the variability of solar wind streams detected by spacecraft close to the Sun as they traverse multiple sources (CHs and ARs) on sufficiently short temporal scales (hours) and small spatial scales (hundreds of kilometres). This variability is often lost at large heliocentric distances due to transport processes.

Here we report the solar wind sources and variability detected by SO between 1 March 2022 and 9 March 2022. We combine ballistic backmapping and spectroscopic analysis techniques together with unique remote-sensing observations and in situ measurements to trace plasma detected by SO from a close distance of ~0.5 au back to its solar sources. We show through the properties and variability of the in situ plasma that the solar wind still exhibits, at this distance, the footprint of its various source regions.

## Results

**Fig. 1:**
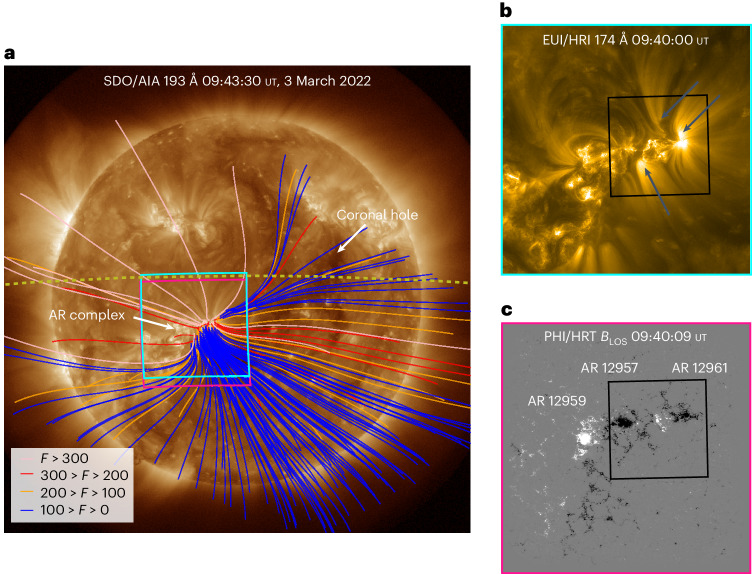
Remote-sensing observations of the predicted slow wind source region. **a**, SDO/AIA 193 Å image showing the source region from the perspective of an Earth observer. Open magnetic field lines that are constructed from the coronal potential field model are overplotted, coloured by their associated expansion factor *F*. The large equatorial CH and AR complex are labelled in white. The FOVs of SO EUI/HRI and PHI/HRT are shown in cyan and pink, respectively. The back-projected trajectory of SO from 1 March 2022 until 9 March 2022 is shown by the olive dotted line (from right to left). **b**, 174 Å image of the AR complex taken by EUI/HRI onboard SO. The blue arrows indicate the locations of upward-propagating features along the coronal fan loops. The black box indicates the FOV of Supplementary Video 1, which has a length of 48 s. The video shows the period 09:40 ut to 10:40 ut on 3 March 2022 with a 5 s cadence. Multiscale Gaussian normalization was applied to sharpen faint structures in the individual EUI/HRI images (see Methods for details). **c**, The PHI/HRT photospheric line-of-sight magnetic field of the AR complex comprising NOAA ARs 12959, 12957 and 12961, which are labelled in white. Black (white) represents negative (positive) magnetic field polarity, saturated at −500 G (500 G).

During the Slow Solar Wind Solar Orbiter Observation Campaign (06:00 ut on 3 March 2022 until 18:30 ut on 6 March 2022), the trajectory of SO spanned across a large equatorial CH-AR complex, visible in 193 Å by the Atmospheric Imaging Assembly (AIA) onboard the Solar Dynamics Observatory (SDO; Fig. 1a). Flux continuously emerged in this complex, leading to the formation of three ARs on 4 March 2022, classified by the National Oceanic and Atmospheric Administration as ARs 12957, 12959 and 12961.

The most striking features of this AR complex are the long-lived, large-scale, coronal fan loops, visible in 174 Å as measured by the Extreme Ultraviolet Instrument High-Resolution Imager (EUI/HRI) onboard SO (Fig. 1b). The fan loops are associated with the leading negative polarities of ARs 12957 and 12961, as shown by the photospheric line-of-sight magnetic field measured by the Polarimetric and Helioseismic Imager High Resolution Telescope (PHI/HRT) onboard SO (Fig. 1c). The loops extend outwards from the negative polarities into the corona. Although lower-temperature fan loops will ultimately be closed, a large fraction of higher-temperature loops will be associated with upflows and potentially open magnetic fields^38^, as confirmed by the potential field extrapolation. Extreme ultraviolet brightenings and upward-propagating features are observed at the base and along these fan loops (Fig. 1b, blue arrows). This suggests that plasma upflowing along these fan loops associated with the open magnetic field could ultimately contribute to the solar wind measured by SO, if the spacecraft is magnetically connected to this region.

**Fig. 2:**
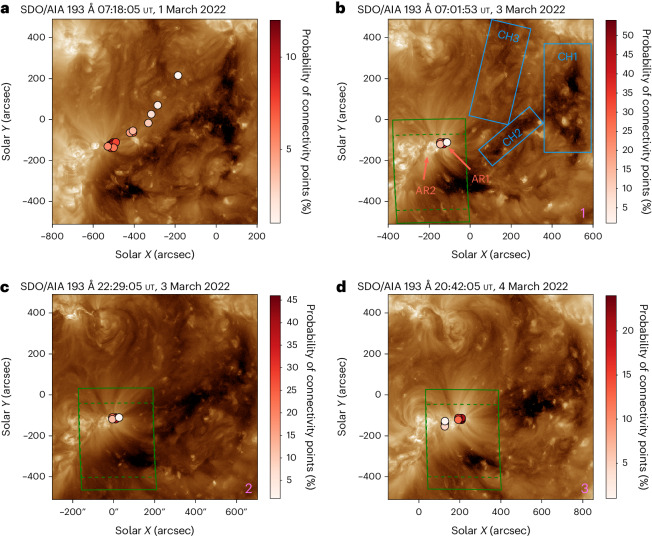
Evolution of the post-observation magnetic connectivity footpoints of SO. **a**–**d**, Connectivity footpoints provided by the magnetic connectivity tool^39^ overlaid on SDO/AIA 193 Å data at four different times both before and during the SO remote-sensing observation window. The connectivity points correspond to a spacecraft (in situ) time of 06:00 ut on 3 March 2022 (**a**), 06:00 ut on 5 March 2022 (**b**), 18:00 ut on 5 March 2022 (**c**) and 12:00 ut on 7 March 2022 (**d**), using solar wind speeds measured by SWA/PAS of 533, 504, 455 and 343 km s^−1^, respectively. The probability of the connectivity points as a percentage is given by the colour bar. The green boxes represent the FOV of the Spectral Imaging of the Coronal Environment (SPICE) instrument. The green dashed boxes show the SPICE FOV with the bright dumbbell removed. The CH1–3 and AR1 and AR2 labels in blue and red in **b** correspond to the probable origins of the different fast and slow solar wind streams originating from sections of the CH and the two ARs.

Modelling the SO connectivity using the magnetic connectivity tool^39^ (Methods) shows that the post-observed connectivity footpoints transition from the dark channel, which merges with the large equatorial CH, to the two ARs. The magnetic connectivity tool provides the estimated solar wind source location at the surface, which is analysed in comparison with the observations to determine the true sources of the solar wind plasma arriving at SO. The labels in Fig. 2 indicate the probable sources of the three fast (CH1–3) and two slow (AR1 and AR2) wind streams detected later in situ by SO that originate from the CH-AR complex.

Initially, on 1 March 2022 (Suntime) (Fig. 2a), the magnetic connectivity footpoints span along CH3 and the leading negative polarity of AR1, with SO marginally more probably connected to AR1. Two days later, on 3 March 2022 (Fig. 2b,c), SO was solely connected to the leading negative polarity of the AR complex (AR1). By 4 March 2022 (Fig. 2d), the footpoint predictions are split across the two leading negative polarities of the AR complex (AR1 and AR2). According to the tool, solar wind plasma originating from the AR complex should be detected in situ at SO from 06:00 ut, 3 March 2022, until 12:00 ut, 7 March 2022 (spacecraft time), giving a travel time of approximately two to three days. Note that although the tool shows that SO was connected to CH3 but not to CH1 or CH2, three fast streams were detected later in situ, with no other plausible solar sources for these streams visible on the Sun.

The composition of the solar wind streams that SO transited through changes with the connectivity to different source regions. Spectroscopic data from SPICE allows characterization of the plasma composition in parts of the AR complex. The results show composition differences, indicating a change in the properties of the potential source regions. The field of view (FOV) of the SPICE rasters from 3 March 2022 and 4 March 2022 are shown in the green boxes in Fig. 2b,c,d.

**Fig. 3:**
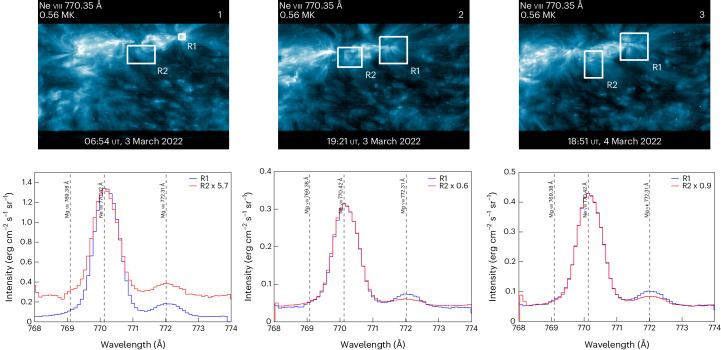
SPICE observations from rasters taken at 06:54 ut on 3 March 2022, 19:21 ut on 3 March 2022 and 18:51 ut on 4 March 2022. The white boxes show the regions where the Mg/Ne abundance ratio was examined. These correspond to the base of the coronal fan loops of ARs 12961 (R1) and 12957 (R2). The spectra are averaged within the boxes and adjusted to emphasize the differences in the Mg viii 772.31 Å intensities. The white numbers in the top right refer to the SPICE raster FOVs shown in Fig. 2.

Figure 3 shows SPICE spectra in a wavelength interval containing critical Mg viii 769.38 Å, Mg viii 772.31 Å and Ne viii 770.42 Å abundance diagnostic spectral lines. The spectra are averaged in regions R1 and R2, using the rasters taken at 06:54 ut and 19:21 ut on 3 March 2022 and at 18:51 ut on 4 March 2022. The boxes encompass the negative polarities of ARs 12961 (R1) and 12957 (R2) and the associated magnetic connectivity footpoints (Fig. 2).

The Mg viii lines are relatively strong compared to the Ne viii lines when the FIP effect−the enhancement of low first ionization potential (FIP; <10 eV) elements in the corona−is operating. By examining the Mg viii 772.31 Å and Ne viii 770.42 Å lines in the two regions at different times, a larger intensity ratio, suggestive of a stronger FIP effect and indicating coronal or above coronal composition, developed at the base of the fan loops associated with the leading negative polarity of AR 12961 (R1). In contrast, the area associated with AR 12957 (R2) showed a smaller intensity ratio, indicative of a weaker FIP effect and suggesting photospheric or weak coronal composition. Figure 3 contrasts the spectra in R1 and R2 at the three different times. The R2 spectra have been adjusted so that the strengths of the Ne viii lines are matched to emphasize where the Mg VIII line is stronger. It is unclear in the concentrated area chosen for R1 at ~7:00 ut (left panel) but is obviously stronger in R1 than R2 in the wider areas selected later (Fig. 3, centre and right panels).

Although the composition can vary substantially between different AR features, individual structures generally maintain the same composition for extended periods. We, therefore, expect that a more detailed emission measure (EM) analysis of these regions (Methods) should give the same result as the line ratio analysis and provide further confidence in our results. All our investigations indicate a clear spatial difference in plasma composition between extended regions R1 and R2. This result is not surprising, as the leading negative polarity of AR 12961 (R1) was already present when the region rotated onto the solar disk and additional magnetic flux began to emerge in this region. Conversely, the second negative polarity started to emerge only on 28 February 2022, a few days before the SO observations, so the enhanced coronal composition had less time to develop^40^.

**Fig. 4:**
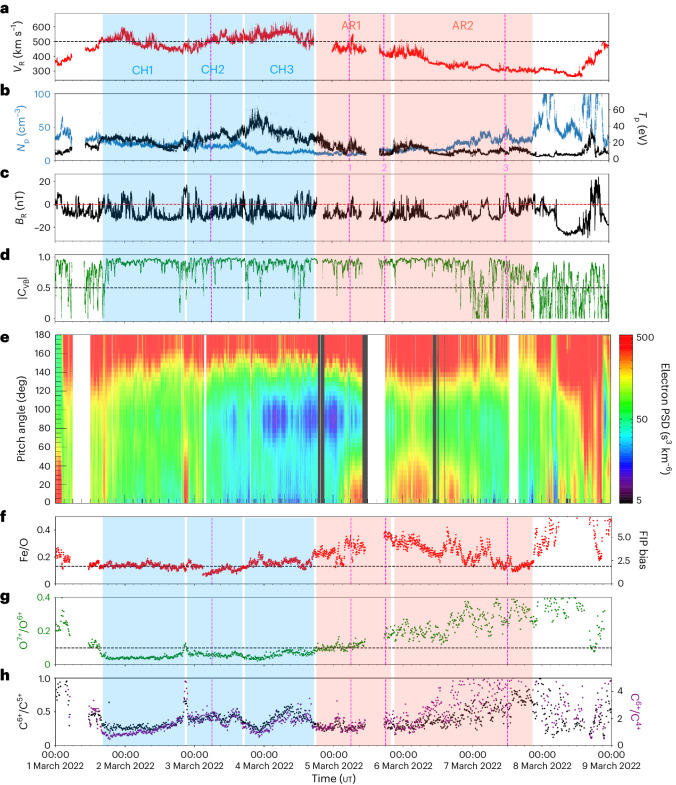
In situ measurements taken from 1 March 2022 to 9 March 2022 by the SWA and MAG instruments onboard SO. **a**,**b**, Radial proton velocity *v*_R_ (red), proton number density *N*_p_ (blue) and proton temperature *T*_p_ (black), as measured by SWA/PAS. The black dashed line in **a** represents a velocity of 500 km s^−1^. **c**, Radial component of the interplanetary magnetic field *B*_R_ (nT) measured by MAG. The red dashed line signifies 0 nT. **d**, Magnitude of the VB correlation factor (green). The black dashed line indicates a correlation factor of 0.5. **e**, Pitch angle distribution of electrons measured by SWA/EAS. The colour shows the electron phase space density (PSD). **f**, Fe/O ratio (red). The corresponding FIP bias values are given on the right *y* axis. The black line signifies a value of 0.13 (refs. ^69,70^). **g**, Charge state ratio for O^7+^/O^6+^ (green). The black dashed line represents a value of 0.145 (ref. ^79^). **h**, Charge state ratios for C^6+^/C^5+^ (black) and C^6+^/C^4+^ (purple). Three fast wind streams (two slow wind streams) are shaded in blue (red) and are labelled CH1, CH2 and CH3 (AR1 and AR2). These originate from the linked sections of the large equatorial CH and the two ARs that are labelled in Fig. 2b. The four magenta dashed lines and three numbers represent the times corresponding to the post-observation magnetic connectivity analysis shown in Fig. 2. The reversals of the radial magnetic field in **d** occurred during the following periods: 15:00–19:00 ut on 1 March 2022, 01:00–03:00 ut, 07:00–11:00 ut and 18:00–22:00 ut on 2 March 2022, 07:00–17:00 ut on 4 March 2022, 02:00–08:00 ut on 6 March 2022, and 03:00–21:00 ut on 7 March 2022.

From 1 March 2022 to 9 March 2022, the solar wind detected by the Solar Wind Analyser (SWA) and the Magnetometer (MAG) onboard SO had varying characteristics, indicating streams originating from multiple sources (Fig. 4). The variations in solar wind properties, as measured by these instruments, are consistent with the composition analysis, SO trajectory and connectivity footpoint track across the CH-AR complex.

Between 1 March 2022 and 4 March 2022, three fast solar wind intervals (blue shaded regions CH1–3 in Fig. 4a) arrived at SO, as evidenced by the peaks in proton velocity above 500 km s^−1^ (Fig. 4a) and the increase in proton temperature (Fig. 4b). The radial magnetic field measured by MAG (Fig. 4c) was directed towards the Sun, consistent with the predominantly negative polarity of the large equatorial CH. The fast wind intervals were followed by an Alfvénic slow wind on 4 March 2022 and 5 March 2022 (Fig. 4a, AR1), both of which are characterized by a strong correlation between the normal component of the velocity (V) and magnetic field (B) vectors, as evidenced by the VB correlation factor (∣*C*_VB_∣, Fig. 4d). The high VB correlation periods during the Alfvénic slow wind interval are accompanied by a low compressibility (almost constant density, *N*_p_), radial velocity (*v*_R_) and magnetic field (*B*_R_) fluctuations.

Another slow wind stream (Fig. 4a, AR2) with velocities ~350 km s^−1^, increasing and highly variable proton densities, and decreasing proton temperatures was detected by SO from 6 March 2022 onwards. Large VB correlation factor fluctuations in this period indicate a poor Alfvénicity correlation. Late on 7 March 2022, an interplanetary coronal mass ejection (ICME) arrived at SO. It originated from an AR complex in the northern hemisphere around 22:30 ut on 6 March 2022. It is identifiable from the sharp discontinuity and subsequent smooth variation in the radial magnetic field and the drastic changes in proton density and temperature.

The VB correlation factor patterns, accompanied by switchback episodes, strongly support the connectivity of SO transitioning across multiple source regions. Several intervals in the VB correlation factor exhibit inverted U shapes formed by drops in this parameter to <0.5 on hourly time scales, bounding longer periods of solar wind plasma with high Alfvénic content. These VB correlation factor depressions often coincided with magnetic field reversals, which may indicate patches of switchback activity, the timings of which are given in the caption of Fig. 4.

Further evidence supporting our magnetic connectivity picture is shown by the strahl electrons detected by the SWA/Electron Analyser System (EAS), Fig. 4e, as they provide a direct probe of the connectivity of SO to the corona^41^. The strahl population manifests as an enhanced suprathermal electron beam (higher phase space densities in Fig. 4e), which appears at pitch angles near to 0° or 180° or both. For most of the period, the dominant magnetic field polarity was inward (*B*_R_ < 0), whereas the enhanced strahl fluxes were predominantly near 180°. The strahl beam was, thus, mostly directed outward during the period shown, consistent with these electrons streaming outward from the corona, antiparallel to the magnetic field direction. An obvious departure from this was in the interval beginning before 02:30 ut, 1 March 2022, when the strahl population appeared near 0°. This was in a region before a heliospheric current sheet crossing, where the dominant magnetic polarity was directed outward, consistent with the outward streaming electrons being parallel to the magnetic field direction.

However, there are numerous other short-term changes in the electron strahl (Fig. 4e). These are associated with the transitions between the solar wind stream boundaries within the CH sections (blue shaded regions, CH1–3) and the AR complex (red shaded regions, AR1 and AR2). Beginning approximately at 17:00 ut, 1 March 2022, and marking the start of the period labelled CH1, there was a short reversal in the radial magnetic field. The strahl beam at 180° dropped out and was replaced by a weaker beam at 0°. This strahl beam switch is inconsistent with expectations for a switchback but indicates a brief connection with an opposite-polarity region.

The CH1/CH2 boundary is also marked by a radial magnetic field reversal at 21:00 ut, 2 March 2022. In this instance, there was no strahl beam dropout at 180° but the additional appearance of a (slightly weaker) beam at 0°. These bidirectional strahl beams may indicate a closed magnetic field (with both magnetic field footpoints connected to the Sun), a small flux rope or electron reflection beyond SO. The persistence of the 180° is also consistent with this reversal being part of a switchback. The electron pitch angle distribution remained strongly anisotropic, with fluxes peaking near 180° in the interval between 10:00 ut on 3 March 2022 and 03:00 ut on 5 March 2022. This was followed by a long bidirectional strahl interval beginning at 03:00 ut on 5 March 2022, suggesting a connectivity transition across the AR complex (Fig. 2b, AR1 and AR2). At 20:00 ut on 6 March 2022, the strahl was predominantly aligned antiparallel until it became more isotropic in association with the ICME arrival at 21:00 ut, 7 March 2022. Note that similar interpretations of these electron signatures were made in ref. ^42^.

The SWA/Heavy Ion Sensor (HIS) measurements (Fig. 4f–h) are also consistent with the spatio-temporal changes in the solar source regions. From the outset, the Fe/O ratio and C and O charge state ratios were low, meaning that SO had detected a plasma with photospheric abundances and temperatures consistent with a CH source. The ratios remained depleted until 14:00 ut, 4 March 2022, except for a small increase at 21:00 ut, 3 March 2022, that coincided with the short burst of bidirectional strahl, magnetic field reversals and large Alfvénic fluctuations. Apart from the C charge state ratios, the other ratios began to increase from 14:00 ut on 4 March 2022 onwards, suggesting that SO had detected solar wind plasma with a coronal composition of higher temperatures, probably from an AR source. This coincided with high Alfvénicity and bidirectional electron strahl. After a data gap on 6 March 2022, the Fe/O ratio dropped to photospheric levels on 7 March 2022 and increased rapidly again before the ICME arrival. Both charge state ratios remained increased and highly variable.

The solar wind velocity decrease is consistent with the inverse relationship between the magnetic field expansion factor and the velocity. Figure 1a shows the open magnetic fields (colour-coded by expansion factor) in the regions CH3, AR1 and AR2 (labelled in Fig. 2). As the magnetic connectivity of SO transitioned from the final CH section (CH3) through the AR polarities (AR1 and AR2), the expansion factor increased notably, thus accounting for the solar wind velocity decrease measured by the SWA/Proton Alpha Sensor (PAS) from 4 March 2022 to 7 March 2022 (Fig. 4). Highly Alfvénic slow wind is often observed due to the over-expansion of magnetic field lines^18,43,44^, such as that exhibited by the core of the AR complex.

## Discussion

Combining the SO trajectory, coronal field model, magnetic connectivity tool, the SPICE composition analysis of the AR complex, and the in situ plasma and magnetic field parameters, we suggest that SO was immersed in three fast wind streams (Fig. 4a, CH1–3) originating from the three linked sections of the large equatorial CH (Fig. 2b, CH1–3). These were followed by two slower streams (Fig. 4a, AR1 and AR2) associated with the negative polarities of the AR complex (Fig. 2b, AR1 and AR2). The decrese of the solar wind speed can be explained by the expansion of the open magnetic field associated with the CH-AR complex, as the connectivity of SO transitioned across these regions.

Three fast solar wind intervals (Fig. 4a, CH1–3) initially arrived at SO between 1 March 2022 and 4 March 2022 (Fig. 4). The three streams are identified by the peaks in solar wind velocity (>500 km s^−1^), proton temperature and high Alfvénicity intervals, as SO passed through solar wind plasma originating from the different sections of the large equatorial CH. The low values of the Fe/O, C and O ratios indicate photospheric abundances and temperatures. The CH1–3 transitions are also evident through the inverted U shapes in the VB correlation factor^44^, as the Alfvénicity was lost due to magnetic sector crossings, along with the bidirectional strahl bursts and charge state ratio spikes. The bidirectional electrons could have been due to a small, ejected flux rope near the boundary of CH1 and CH2, with the small increase in the Fe/O ratio due to plasma confinement in a closed magnetic field before the eruption. The increase in the O and C charge state ratios could be due to extra coronal heating, as the flux rope was formed by reconnection during the eruption. The flux rope could be formed by a magnetic field reversal like the scenario in refs. ^6,41^, for which the photospheric footpoints of the open magnetic field cross the CH1/CH2 boundary, leading to a sizeable change in the plasma velocity. This would result in the formation of a heliospheric magnetic field reversal through interchange reconnection, like that in Fig. 8 of ref. ^6^.

There was a clear fast to Alfvénic slow wind transition beginning 4 March 2022 (Fig. 4, AR1), with decreasing proton temperature, high VB correlation, and increasing values of Fe/O and O charge state ratios, indicating coronal AR abundances and temperatures. The changes seen in O^7+^/O^6+^ but not in the C charge state ratios reflect coronal temperature profile changes. These correlate well with the post-observation magnetic connectivity predictions that suggest that SO was magnetically connected to the leading negative polarity of AR 12961 and the analysis of the Mg/Ne abundance ratio, which suggests a strong FIP effect (coronal abundance) in this region (Fig. 3). The bidirectional strahl present during 5 March 2022 to 7 March 2022 indicates that the magnetic field was locally connected back to the Sun, which was not the case for most of the interval. There could have been a closed expanding loop associated with the emerging AR complex, which is indicated by the slight asymmetry of the bidirectional strahl as the magnetic field lines mapped back to different points on the Sun.

SO then entered a slow wind from 6 March 2022 to 8 March 2022 (Fig. 4a, AR2) with poor Alfvénic correlation along with photospheric abundances and highly variable charge state ratios. Along with the magnetic connectivity and the SPICE composition analysis of the Mg/Ne abundance ratio, the in situ data suggest that SO sampled the solar wind originating from the second negative polarity of the AR complex, where a weaker FIP effect (photospheric abundance) was detected. Therefore, the two slow streams (Fig. 4, AR1 and AR2) probably originated from the negative polarities of ARs 12961 and 12957, as the connectivity of SO changed across the two regions (Fig. 2a, AR1 and AR2).

Solar wind plasma must stream along open field lines if it is to be released into the heliosphere. Therefore, we propose that coronal plasma contained in the core closed field of the leading AR (AR1) escaped along open fan loops associated with the AR and that this could occur through interchange reconnection. This interchange reconnection scenario is supported by the over-expanding magnetic field due to the continuous emergence of the second AR (AR2). The emerging bipoles created favourable sites for interchange reconnection between open-closed flux systems. This scenario is further supported by the asymmetrical bidirectional electron strahl and the arrival of highly Alfvénic slow wind.

Coronal field and magnetic connectivity modelling, remote-sensing elemental composition, and in situ measurements of solar wind velocities, proton densities and temperatures, radial interplanetary magnetic field, electron pitch angle distributions, heavy-ion charge state ratios, and Alfvénicity all support the outlined scenario. By utilizing these remote-sensing and in situ SO datasets, along with magnetic modelling and spectroscopic techniques, we have advanced our understanding of how the variability and complexity of solar wind detected in situ is driven by changes in the magnetic connectivity and evolution of source regions in the solar corona. The macroscale variability of the stream structure measured in situ, which can be caused by the changing magnetic connectivity of a spacecraft as it traverses multiple source regions, is expected to be ubiquitous. Therefore, these results are relevant for other heliospheric observatories.

## Methods

The data presented and analysed here were taken during SO’s first perihelion passage as part of the Slow Solar Wind Connection Science SO Observing Plan^45,46^. Remote-sensing instruments took observations between 06:00 ut on 3 March 2022 and 18:30 ut on 6 March 2022, when SO was at a radial distance [0.55, 0.51] au with a separation angle of [7°, 3°] from Earth. Continuous in situ measurements were taken before, throughout and after this time window.

For this article, we utilized remote-sensing observations and in situ measurements from SDO/AIA, the Global Oscillations Network Group (GONG) and SO (EUI, PHI, SPICE, SWA and MAG), along with the magnetic connectivity tool and composition analysis techniques to characterize multiple streams of solar wind plasma detected by SO in the heliosphere and to link them back to their coronal sources.

### Remote-sensing observations

The SDO/AIA 193 Å image were obtained online through the Science Data Processing database at the Joint Science Operations Center hosted by Stanford University. The AIA instrument^47^ onboard SDO^48^ provides full-disk images of the solar atmosphere in multiple wavelength channels with a 1.5" spatial resolution and 12 s temporal cadence.

To calculate the magnetic field expansion factor for the open magnetic field lines rooted in the active region complex and the surrounding area, we constructed a potential field source surface (PFSS) extrapolation using a synoptic photospheric magnetic field map from GONG.

The back-projected trajectory of SO between 1 March 2022 and 9 March 2022 was plotted by retrieving the position of SO using the SPICE kernel from HelioPy (10.5281/zenodo.5903184) and using the PFSS extrapolation, along with solar wind speeds measured in situ by SWA/PAS.

The GONG synoptic map was downloaded from the National Solar Observatory’s data archive (https://gong.nso.edu/data/magmap/QR/zqs/). To derive near-real-time, hourly synoptic maps of the Sun, full-disk, 2.5" pixel images of the photospheric field with a noise level of 3 G were taken at six different ground sites, each minute, 24 hours a day. The images were averaged and corrected for the annual periodic modulation in the polar regions and remapped into longitude and sine (latitude). To obtain flux densities, the line-of-sight component of the magnetic field was converted by assuming a radial magnetic field.

The synoptic map chosen to construct the PFSS extrapolation was last updated at 10:04 ut on 3 March 2022, very close in time to the SDO/AIA 193 Å image shown in Fig. 1. In total, 9,150 magnetograms were used to construct the synoptic map. The map was then loaded into Python using the SunPy package (https://sunpy.org/), and the mean radial field was subtracted. The pfsspy package^49^ was used to calculate the PFSS solution in spherical coordinates by using a 40 × 40 grid of seed points to trace the magnetic field out to a source surface of 2.5 *R*_⊙_. We used the SunPy package to plot the resulting field lines that are considered open at the source surface on the SDO/AIA 193 Å image. The open field lines are colour-coded by the magnetic field expansion factor, which was calculated between 1 and 2.5 *R*_⊙_ using $F(R)=({R}_{\odot }^{2}{B}_{\odot })/({R}_\mathrm{ss}^{2}{B}_\mathrm{ss})$ where *R* is the radial distance, *B* the magnetic field strength and ss represents the source surface.

All SO data used here are publicly available through the ESA SO Archive (SOAR; https://soar.esac.esa.int/soar/#home).

The 174 Å image of the AR complex was taken by EUI/HRI^50^. Level 2 FITS files taken from EUI Data Release 6 (ref. ^51^) were used in this work (10.24414/z818-4163). These were plotted in Python using the SunPy package. EUI/HRI has a pixel size of 0.492" and a FOV of 16.8' × 16.8'. The 174 Å waveband is sensitive to and observes plasma from the low corona with a peak temperature of around 1 MK. Supplementary Video 1 was created using level 2 FITS files from EUI Data Release 6 and covers the period from 09:40 ut until 10:40 ut on 3 March 2022 with a temporal cadence of 5 s. The level 2 FITS files have already been processed using the EUI data-processing pipeline and are suitable for scientific analysis. The images were then further processed using the multiscale Gaussian normalization technique of ref. ^52^ to highlight faint structure and enable a detailed analysis of the observed region of interest.

The line-of-sight component of the photospheric field was taken by PHI/HRT^53^, which has a FOV of 0.28° × 0.28° and a pixel size of 0.5". Aberrations that were introduced by the nonradial temperature gradient in the entrance window were removed^54^. The level 2 FITS file was downloaded from SOAR, and the image was plotted using the SunPy package in a similar manner to the EUI/HRI 174 Å image.

The SPICE data^55^ (10.48326/idoc.medoc.spice.2.0) we analysed were downloaded from SOAR. We used one full detector spectral atlas and three raster observations. The SPICE observation IDs are 10663695, 10663696, 10663698 and 10663704. The atlas observations contain spectra over the full wavelength ranges (704–790 Å and 973–1,049 Å), whereas the other three rasters telemeter a subset of spectral lines to the ground. All the datasets were for the 4" slit with ~60 s exposures. The atlas observations covered a FOV of ~133" × 1,080" in around 51 min, whereas the raster observations covered an FOV ~636" × 915" in around 2 h 40 min. Details of the SPICE instrument are available in refs. ^55,56^.

### Magnetic connectivity tool

We used the magnetic connectivity tool to estimate the coronal source region of the solar wind plasma that was detected in situ by SO (ref. ^39^, http://connect-tool.irap.omp.eu/). The connectivity tool combines different techniques to model both the heliospheric and coronal magnetic field to establish the magnetic connectivity of a spacecraft to the solar surface.

In this particular case, the tool assumed the heliospheric magnetic field to be a Parker spiral where the shape of the spiral was determined by the radial solar wind velocity measured by SWA/PAS, when these measurements are available. To reconstruct the coronal magnetic field, the tool used the PFSS model^57,58^ using ADAPT (Air Force Data Assimilative Photospheric Flux Transport, https://gong.nso.edu/adapt/maps/) synchronic synoptic maps as the input boundary conditions. ADAPT uses an ensemble of magnetic field maps and data assimilation techniques^59^ to provide a realistic representation of the global photospheric magnetic field. When no magnetic field observations are available, ADAPT uses a flux transport model^60^ to evolve active regions. The ADAPT maps are publicly available (https://www.nso.edu/data/nisp-data/adapt-maps).

The connectivity tool must compute the solar wind release time to determine the appropriate ADAPT synchronic map as input to reconstruct the coronal magnetic field. The release time is defined by the chosen propagation mode, which we selected as ‘spacecraft to Sun’ with ‘SW (solar wind) lag’, as we traced the origins of solar wind plasma detected in situ by SO back to its source region on the Sun.

The connectivity points were determined by tracing the intersection of SO with the Parker spiral down to the source surface (the upper boundary of the PFSS model where the field lines are forced to be radial), then down to the solar surface in the coronal model. The tool outputs a distribution of connectivity points at the surface, which was produced by sampling an uncertainty ellipse around the position of SO and tracing multiple field lines across the heliospheric and coronal field. The position and probability of the connectivity points obtained from the connectivity tool were then overplotted on SDO/AIA 193 Å images that correspond with the timings of the ADAPT synchronic maps.

The simple approach of using a PFSS magnetic field extrapolation along with the Parker spiral assumption based upon measured solar wind speed has limitations. However, it is a still a powerful tool that requires minimal computational time. The potential field assumption is representative of the corona only during quiet periods of solar activity. In our case, the large-scale coronal field of the active region complex was not observed to change substantially during the period studied.

### SPICE composition analysis

We examined in the main text the Mg/Ne abundance variations using the simple Mg viii 772.31 Å/Ne viii 770.42 Å line intensity ratio. Although the temperature responses of the Mg viii and Ne viii lines are similar, they are not identical, so it is important to confirm that the composition measurements we made were not impacted by temperature variations. Ideally, we would determine the electron density and compute the temperature distribution in the regions analysed so that we could more accurately model the intensities of this diagnostic ratio. However, the rasters analysed do not have sufficient spectral lines of enough elements to perform this analysis. Therefore, we underpin these results with a more complete EM analysis of the plasma composition of the wider regions R1 and R2 using full detector spectra taken 1 h before the first raster. As noted in the main text, the plasma composition in the observed structures is expected to be similar on this timescale.

Furthermore, for the EM analysis, we applied the photometric calibration to convert the count rates to physical units. The SPICE data are calibrated in units of W m^−2^ sr^−1^ nm^−1^, and we converted these to cgs units (erg cm^−2^ s^−1^ sr^−1^ Å^−1^). As the uncalibrated data are noisy and the Mg viii 772.31 Å line is weak, correcting the spectra also means that this feature emerges more prominently from the background. However, we also examined the line profile counting statistics to determine whether the variations we detected between R1 and R2 in Fig. 3 are significant. The uncalibrated spectra are very noisy for the first dataset (Fig. 3, left panel), and we concluded that the results for the small R1 box are ambiguous. For the other datasets (Fig. 3, middle and right panels), the counting error for the Mg/Ne intensity ratio is 12.9%–17.3%. The differences in the ratios, however, are factors of 1.8–2.2 larger, indicating that they are significant. In the future, we hope that new observation campaigns will be developed that potentially lead to full spatial composition maps of target regions.

For our EM analysis, we followed the basic methodology of ref. ^61^. Line intensities were obtained by fitting single or multiple Gaussian functions to the spectra, as appropriate for clean or blended lines. We identified the lines using the SOHO/SUMER higher-resolution spectral atlas^62^. An approximately 25% error was adopted to account for the photometric calibration uncertainty. In the analysis of line intensities, this uncertainty dominates over other sources of error. For example, the spectra analysed for the EM analysis are averages of the spectra within the boxed areas shown in Supplementary Fig. 1. These boxes contain at least 1,300 pixels, implying a counting error of less than 3%. This had a minimal impact when added in quadrature to the photometric calibration uncertainty. The line profile counting statistics discussed above also had a minimal impact on this analysis.

We used lines of O iii–vi, S iv–v, N iv, Ne vi and viii, and Mg viii and ix for the EM analysis. These cover a range of temperatures from 0.5 to 1.0 MK. The exact lines are given in Supplementary Table 1 along with our results, and the EM solutions are shown in Supplementary Fig. 2.

We used the Markov-chain Monte Carlo (MCMC) algorithm in the PintOfAle^63,64^ SolarSoftWare package^65^ to compute the EM distribution and the CHIANTI database^66^ v.10 (ref. ^67^) supplemented with ADAS (ref. ^68^) to compute the contribution functions (*G*(*T*, *n*)) for each spectral line. The MCMC algorithm performs an inversion using the observed line intensities (*I*) and contribution functions *I* = *A*∫*G*(*T*, *n*)*ϕ*(*T*) d*T*, where *A* is the elemental abundance, *n* the electron density, *T* the electron temperature and *ϕ*(*T*) the differential EM. It finds the best-fitting solution that minimizes the differences between observed and computed intensities from a collection of 100 Monte Carlo simulations. The critical issue is that we assumed values for the abundances *A*, and post-calculation, we verified which assumed abundances worked best. For this work, we adopted three sets of abundances: the photospheric abundances from refs. ^69,70^, the coronal abundances from ref. ^71^ and the coronal abundances from ref. ^72^. We used two sets of coronal abundances to gain some insight into how strong the FIP effect is. The enhancement factor for the low-FIP element Mg is a factor of 2 greater in the abundances from ref. ^72^ than in the dataset of ref. ^71^.

Ref. ^61^ showed the potential for SPICE to make Mg/Ne abundance measurements, but an important point is that they used test data to assess their measurement technique, so the situation was somewhat idealized (because they were able to pick any measurement target in their observations). For solar wind connectivity studies, however, the measurements are more difficult because we have no choice as to the target: we have to try to make the measurement at the predicted connectivity footpoint. For our analysis, the measurement was, therefore, more challenging. For example, the Mg viii 782.34 Å line was weaker in R2, and it was especially weak in our atlas observations. Although our line profile fitting procedure was still able to fit the weak spectral features, in this case that was because the solution was a shallow Gaussian with unreasonably extended wings. This led to an erroneously large observed intensity, which was then difficult for the EM calculation to reproduce, regardless of the assumed abundances (see the results in Supplementary Table 1). The electron density measurement (normally used to calculate the *G*(*T*, *n*) functions) is, therefore, also unsound. To mitigate this issue, we allowed the MCMC algorithm to find the best-fitting density when acquiring the EM solution.

The results in Supplementary Fig. 2 and Supplementary Table 1 show that the different assumptions for the abundances lead to relatively good solutions in all cases. For example, assuming photospheric abundances in R1, 80% of the line intensities were reproduced within 35%. The table also shows the results assuming the coronal abundances of ref. ^72^. In this case, 90% of the lines were reproduced, suggesting a slight improvement. The results for lines from the low-FIP elements (S and Mg) are helpful in showing a clearer difference. Only about 50% of the intensities of lines from the low-FIP elements were reproduced when photospheric abundances were used, whereas 75% of the lines from the low-FIP elements were reproduced when coronal abundances were adopted. The chi squared calculated for the results also improved. Adopting the abundances from ref. ^71^ (not shown in the table) gave intermediate results between the two cases shown. Given the uncertainties, it was difficult to pin down an exact value for the FIP bias (ratio of coronal to photospheric abundances), but for R1, there is a clear trend indicating improvements in the solution as the FIP bias was increased.

For R2, the results are more marginal. The table shows that 80% of the line intensities were reproduced with photospheric abundances. Although chi squared decreased with increasing FIP bias, the number of line intensities reproduced dropped to 70%. The number of intensities from lines of low-FIP elements that were reproduced also decreased. It was, therefore, difficult to conclude whether photospheric or coronal abundances provide a better solution in R2. We can at least conclude that R1 shows a stronger FIP effect than R2 (if it shows any at all). These results are consistent with what we observe when looking at only the Mg viii 782.34 Å/Ne viii 770.42 Å intensity ratios in the two regions and support the interpretation of the spectra of these regions in the other more limited raster datasets discussed in the main text. Also note that changing the box size between the rasters, particularly for R1, did not affect the results.

### In situ measurements

For this study, data were utilized from all three sensors of the SWA suite (10.5270/esa-ahypgn6)^73^. The radial solar wind velocity (*V*_R_, km s^−1^), proton number density (*N*_P_, cm^−3^) and proton temperature (*T*_P_, eV) shown in Fig. 4a,b were derived from measurements by the SWA/PAS instrument and have a 4 s time resolution. These parameters were extracted into Python from the ground moment level 2 data files available from SOAR using the SpacePy pycdf module^74^. Note that the velocity, density and temperature moments calculated on the ground from the measured velocity distribution function may have been affected by reduced counting efficiencies for the lowest energies in the velocity distribution function. This issue may arise during intervals of particularly low solar wind velocity, typically in the range 260–380 km s^−1^. A data quality indicator is provided for this data (Supplementary Fig. 3), and for most of the period of interest (1 March 2022 until 7 March 2022), this was low, <0.2, the threshold below which data can be assumed to have good quality. During sporadic periods from 7 March 2022, this issue had some impact on the data, and the quality indicator exceeded this threshold but remained <2. Finally, the quality indicator varied strongly and the upper limit was exceeded briefly during the arrival of the ICME on 7 March 2022. Overall, the SWA/PAS data are of good quality as the data quality indicator remained relatively low during the period considered.

The electron phase space density as a function of pitch angle and summed for energies >70 eV is shown in Fig. 4e. These data were derived from measurements by the dual-head SWA/EAS instrument^73^, which has a 10 s time resolution. The three-dimensional electron velocity distribution from each sensor head is available from SOAR. These data were combined and rebinned with reference to the direction of the prevailing magnetic field measured by the MAG instrument^75^ (and see below) to produce the pitch angle distribution as a function of electron energy for each measurement. A summation over the energy range >70 eV was used here as this range is typically dominated by the strahl component of the solar wind electron distribution, a generally field-aligned beam that can be used to infer the magnetic connectivity of the solar wind at the spacecraft to the corona.

The Fe/O, O^7+^/O^6+^, C^6+^/C^5+^ and C^6+^/C^4+^ ratios were produced at a 10 min time cadence from measurements by the SWA/HIS instrument. Additional details on the production of these data products can be found in ref. ^76^. The level 3 data files were downloaded from SOAR and read into Python using the cdflib module (10.5281/zenodo.7011489). The FIP bias values shown on the right-hand axis of Fig. 4f were calculated by taking the Fe/O ratio and dividing by the photospheric value of 0.064 (ref. ^77^).

The radial component of the interplanetary magnetic field, measured by the MAG instrument^75^ (10.5270/esa-ux7y320), is shown in radial–tangential–normal coordinates with a 1 min time cadence. The data were downloaded from SOAR and imported into Python using read_cdf in the SunPy package. The quality flag (Supplementary Fig. 3) for the MAG data was between 2 and 3 for the time period. For the first two days (1 March 2022 to 3 March 2022), there was mainly survey quality data followed by publication quality data (from 3 March 2022 onwards).

### VB correlation factor

The interplanetary magnetic field and the solar wind velocity measured by MAG and SWA/PAS, respectively, were used to compute the magnitude of the VB correlation factor (∣*C*_VB_∣) using a 30 min running window, 30 min being a typical Alfvénic scale^78^. The factor was calculated using the normal components of the velocity and magnetic field only. The normal component of the magnetic field in Alfvén units is given by *b*_n_ = *B*_n_/(4π*ρ*)^0.5^, where *ρ* is the mass density.

### Supplementary information

Supplementary Information Supplementary Table 1 and Figs. 1–3.

Supplementary Video 1 Supplementary video that accompanies Fig. 1 in the main text.

## Data Availability

All the data analysed in the manuscript are publicly available online. URL links are provided in Methods. Correspondence and requests for materials should be sent to Stephanie Yardley.

## Code availability

The PFSS code is available in the pfsspy package^49^. The magnetic connectivity tool can be found online at http://connect-tool.irap.omp.eu. The MCMC algorithm used for the EM analysis is available in the PintOfAle SolarSoftWare package. The contribution functions for each spectral line were computed using the CHIANTI database v.10 supplemented with ADAS.
