# Supplementary material for: Multi-source connectivity as the driver of solar wind variability in the heliosphere
Source: Nat Astron. 2024 May 28;8(8):953–63. doi: 10.1038/s41550-024-02278-9 (PMC11335567; doi:10.1038/s41550-024-02278-9)
Supplement: Supplementary file 1 — Supplementary Table 1 and Figs. 1–3. [file 41550_2024_2278_MOESM1_ESM.pdf]

nature astronomy

Supplementary information

https://doi.org/10.1038/s41550-024-02278-9

Multi-source connectivity as the driver of solar wind variability in the heliosphere

In the format provided by the authors and unedited

Supplementary Information

The supplementary file includes:

- Supplementary Figure 1
- Supplementary Table 1
- Supplementary Figure 2
- Supplementary Figure 3

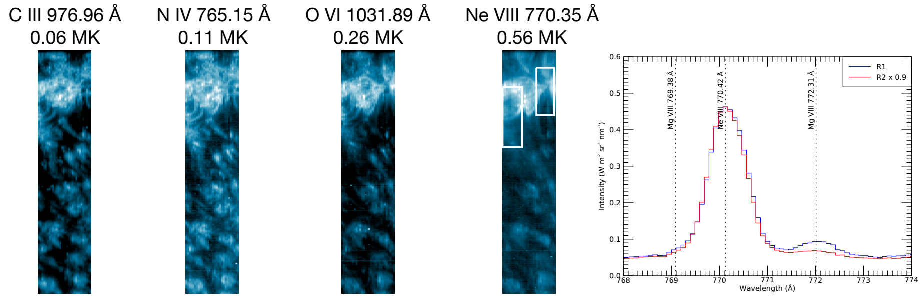

Supplementary Figure 1: Images and spectra from the SPICE spectral atlas data used for the emission measure analysis. Left panel: images summed over the wavelength window with the dominant line indicated along with the formation temperature. Boxes R1 (right) and R2 (left) show the regions where the emission measure analysis was performed. Right panel: average spectra within boxes R1 (blue) and R2 (red) showing the relative strength of the Mg VIII 772.31 Å line. The R2 spectra have been adjusted by a factor of 0.9 to emphasize the difference in Mg VIII 772.3 1Å strength.

Supplementary Table 1: Results of the EM analysis for regions R1 and R2 shown in Supplementary Fig. 1. ID: spectral line identifications. I_{obs}: observed intensities. I_{calc}: calculated intensities. Δ: difference between observed and calculated intensities expressed as a percentage of the observed intensity.

		R1 - Photospheric		R1 - Coronal			R2 - Photospheric		R2 - Coronal	
ID	I_{obs}	I_{calc}	Δ[%]	I_{calc}	Δ[%]	I_{obs}	I_{calc}	Δ[%]	I_{calc}	Δ[%]
O III 702.61	11.8±4.0	8.4	-28.5	7.6	-35.6	13.0±4.3	14.6	11.8	12.8	-1.9
O III 703.87	12.4±4.0	15.5	24.3	13.9	12.0					
Mg IX 706.02	7.2±2.2	6.2	-13.6	7.0	-2.9	5.2±1.7	5.0	-4.8	5.2	-1.1
S IV 748.40	0.8±2.3	0.5	-35.5	0.7	-13.4	1.1±1.1	0.8	-29.7	0.9	-12.9
Mg IX 749.54	1.8±61.3	1.0	-45.4	1.0	-42.3	2.0±30.2	0.8	-62.4	0.8	-61.8
S IV 750.20	2.8±71.6	1.4	-51.5	1.8	-34.9	1.4±34.0	1.8	30.7	2.3	62.0
O V 760.43	13.0±6.1	9.6	-25.6	11.0	-15.3	12.1±5.1	7.1	-41.2	6.7	-44.4
O V 761.99	2.6±1.6	2.7	4.8	3.1	19.2	2.1±1.6	2.3	11.0	2.2	5.1
N IV 765.15	19.8±5.4	21.0	6.1	19.6	-1.2	22.8±6.1	21.5	-5.7	21.0	-8.0
Mg VIII 769.38	0.6±1.0	0.6	1.6	1.3	135.1	0.5±0.9	0.6	15.0	1.0	105.8
Ne VIII 770.42	39.8±10.3	45.2	13.4	43.4	8.9	45.2±11.7	49.6	9.7	48.7	7.7
Mg VIII 772.31	4.2±1.9	1.7	-60.0	3.9	-8.1	2.8±1.9	1.5	-48.3	2.6	-8.2
Ne VIII 780.30	23.2±6.2	22.4	-3.7	21.5	-7.5	25.6±6.8	24.5	-4.1	24.1	-5.9
Mg VIII 782.34	3.7±2.3	1.5	-60.1	3.4	-8.1	118.9±65.8	1.4	-98.8	2.5	-97.9
S V 786.47	13.6±4.2	9.2	-32.3	12.5	-8.6	10.3±3.4	6.8	-33.9	9.8	-4.4
O IV 787.72	23.8±6.6	23.1	-2.9	24.6	3.4	22.6±6.3	21.8	-3.4	21.4	-5.4
O IV 790.11	41.7±10.9	46.4	11.2	49.4	18.5	38.7±10.1	43.2	11.9	42.3	9.5
Ne VI 999.27	5.3±1.9	3.5	-34.7	3.8	-29.4					
Ne VI 1005.79	1.8±1.1	2.2	17.1	2.3	26.7	1.8±1.1	2.0	12.5	1.9	4.0
O VI 1031.93	198.3±50.2	221.0	11.4	189.5	-4.5	176.3±44.6	194.8	10.5	187.7	6.4

SPICE line intensities are in units of erg cm^{-2} s^{-1} $steradian^{-1}$.

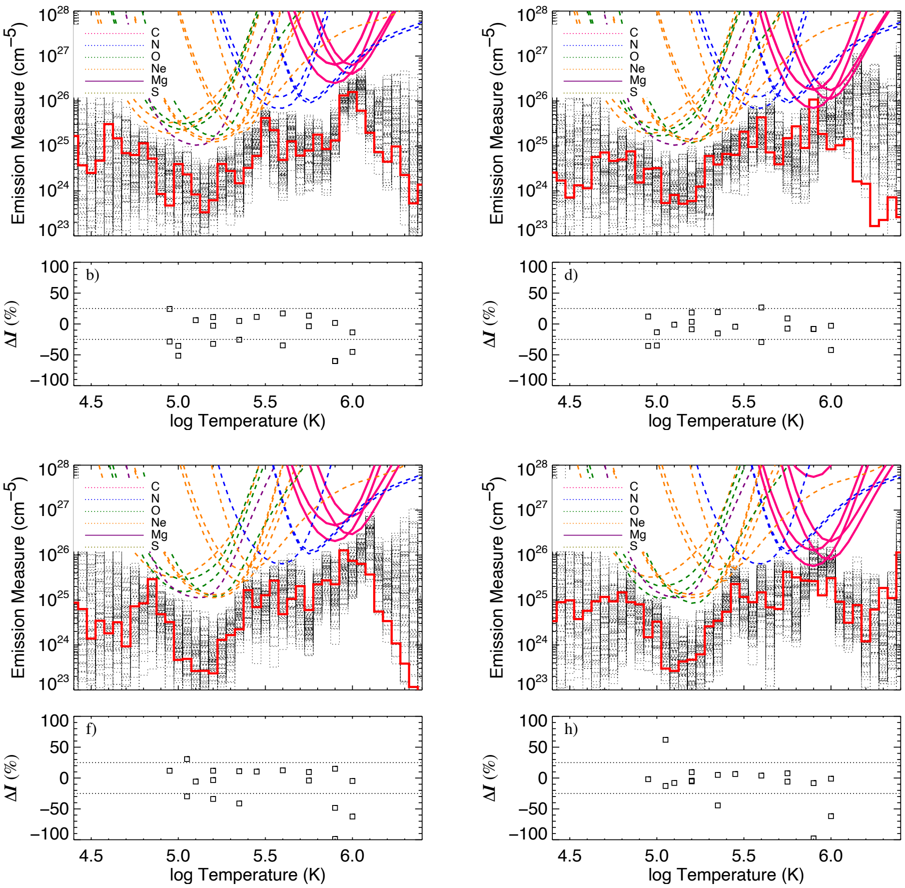

Supplementary Figure 2: Emission measure (EM) analysis of the SPICE observations in regions R1 (a, c) and R2 (e, g) for the full detector spectral atlas observations taken on 2022-03-03 at 06:00 UT. The upper panels show the EM analysis. The left and right columns show the results assuming photospheric or coronal abundances, respectively (see text). The solid red line shows the best fit solution to a collection of Monte Carlo simulations indicated by the dotted gray lines. The other colored lines show emission measure loci curves. These show the upper limit constraints on the emission measure solution, under the assumption that the spectral lines are not detected. The different colors identify the elements used, and these are shown in the legend. The dashed loci curves indicate high-FIP elements and the solid curves indicate low-FIP elements. The lower panels (b, d, f, h) in each quadrant plot the differences between the observed and emission measure calculated intensities as a function of spectral line formation temperature. This gives an indication of which solutions work better - although the detailed numbers are given in Supplementary Table 1. The dotted lines show where the differences would exceed 25% (comparable to the photometric calibration uncertainty).

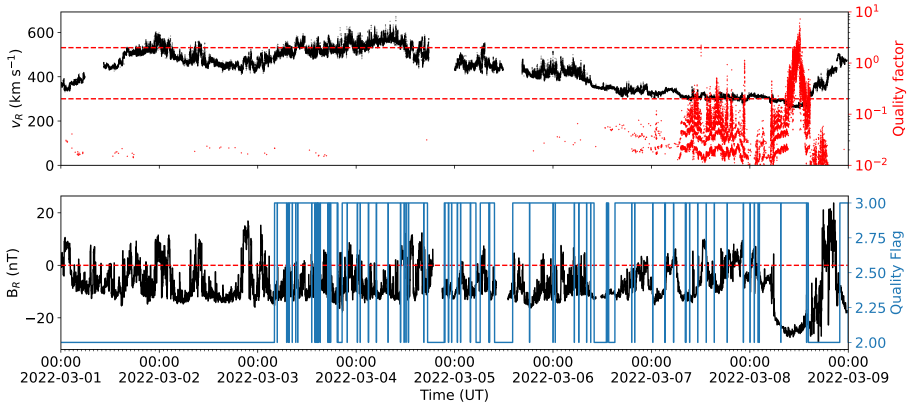

Supplementary Figure 3: The quality factor for the SWA/PAS and MAG data during 2022-03-01 to 2022-01-09. (a) shows the radial solar wind velocity measured by SWA/PAS (black) along with the quality factor (red). The lower (0.2) and upper (2) thresholds are indicated by the dotted red lines. (b) shows the radial magnetic field measured by MAG where the red dashed line represents 0 nT. The associated data quality flag is shown in blue.
